# Linezolid Dose That Maximizes Sterilizing Effect While Minimizing Toxicity and Resistance Emergence for Tuberculosis

**DOI:** 10.1128/AAC.00751-17

**Published:** 2017-07-25

**Authors:** Shashikant Srivastava, Gesham Magombedze, Thearith Koeuth, Carleton Sherman, Jotam G. Pasipanodya, Prithvi Raj, Edward Wakeland, Devyani Deshpande, Tawanda Gumbo

**Affiliations:** a Center for Infectious Diseases Research and Experimental Therapeutics, Baylor Research Institute, Baylor University Medical Center, Dallas, Texas, USA; b Department of Immunology, UT Southwestern Medical Center, Dallas, Texas, USA; c Department of Medicine, University of Cape Town, Observatory, South Africa

**Keywords:** optimal dose, whole-genome sequencing, RNA sequencing, efflux pump regulators, mutations, efflux pumps

## Abstract

Linezolid has an excellent sterilizing effect in tuberculosis patients but high adverse event rates. The dose that would maximize efficacy and minimize toxicity is unknown. We performed linezolid dose-effect and dose-scheduling studies in the hollow fiber system model of tuberculosis (HFS-TB) for sterilizing effect. HFS-TB units were treated with several doses to mimic human-like linezolid intrapulmonary pharmacokinetics and repetitively sampled for drug concentration, total bacterial burden, linezolid-resistant subpopulations, and RNA sequencing over 2 months. Linezolid-resistant isolates underwent whole-genome sequencing. The expression of genes encoding efflux pumps in the first 1 to 2 weeks revealed the same exposure-response patterns as the linezolid-resistant subpopulation. Linezolid-resistant isolates from the 2nd month of therapy revealed mutations in several efflux pump/transporter genes and a LuxR-family transcriptional regulator. Linezolid sterilizing effect was linked to the ratio of unbound 0- to 24-h area under the concentration-time curve (AUC_0–24_) to MIC. Optimal microbial kill was achieved at an AUC_0–24_/MIC ratio of 119. The optimal sterilizing effect dose for clinical use was identified using Monte Carlo simulations. Clinical doses of 300 and 600 mg/day (or double the dose every other day) achieved this target in 87% and >99% of 10,000 patients, respectively. The susceptibility breakpoint identified was 2 mg/liter. The simulations identified that a 300-mg/day dose did not achieve AUC_0–24_s associated with linezolid toxicity, while 600 mg/day achieved those AUC_0–24_s in <20% of subjects. The linezolid dose of 300 mg/day performed well and should be compared to 600 mg/day or 1,200 mg every other day in clinical trials.

## INTRODUCTION

Due to the emergence of multidrug-resistant tuberculosis (MDR-TB) and extensively drug-resistant tuberculosis (XDR-TB), the oxazolidinone linezolid was investigated in salvage regimens starting more than 12 years ago, while the *in vitro* effect against Mycobacterium species has been known for 2 decades (1, 2). Linezolid is now being used for treatment of X/MDR-TB in adult patients (3–5). Salvage therapy studies demonstrated a striking sterilizing effect rate of linezolid as virtual monotherapy; negative sputum cultures were encountered as early as 2 weeks in some patients and at a median of 2.5 months in all patients (3). However, linezolid-induced neuropathy is encountered in 16%, myelosuppression is encountered in 33%, and other adverse events are encountered in 30% of tuberculosis patients (5). In order to reduce adverse events, linezolid doses of 300 mg and 600 mg per day have been administered (5). Case series have reported use of linezolid at a 300-mg/day dose in MDR-TB patients (6). Since subtherapeutic exposures could lead to acquired drug resistance (ADR) (7), while higher doses could be associated with toxicity, therapeutic drug monitoring was advised to help adjust doses down to 300 mg a day (8). However, it is unclear if these doses are optimal and if ADR would be worse at these lower doses. Studies of the efficacy of linezolid against intracellular Mycobacterium tuberculosis in childhood disease, based on pediatric pharmacokinetics and the hollow fiber system model of tuberculosis (HFS-TB), have identified optimal linezolid doses in regimens that omit all first-line drugs and do not include an injectable agent (9–12). Here, we used HFS-TB for studying the sterilizing effect on adult-type pulmonary cavitary disease using intrapulmonary pharmacokinetics, in order to identify the optimal sterilizing effect dose that would minimize the rate of adverse events (13–19). We focused on sterilizing effect, and not bactericidal effect, which is important for long-term outcomes such as cure and relapse.

When linezolid was used as salvage therapy, more than 10% of XDR-TB patients developed acquired linezolid resistance (3). Linezolid kills M. tuberculosis by binding and blocking tRNA in the peptidyltransferase center (PTC) on the 50S ribosomal subunit, which includes the 5S rRNA and 23S rRNA. Thus, it is not a major surprise that some linezolid-resistant laboratory isolates have mutations in 23S rRNA and the 50S ribosomal protein L3 (*rplC*), which binds to 23S rRNA (2). However, in clinical isolates from patients who failed linezolid-based therapy, all isolates lacked mutations in these genes but had MICs that were decreased by 1 tube dilution by the efflux pump inhibitor reserpine (1). Indeed, the molecular evolution of linezolid ADR in M. tuberculosis is unclear, and whole-genome sequencing (WGS) of resistant M. tuberculosis clinical isolates has not been extensively employed. The HFS-TB offers the advantage of repetitive sampling of the same HFS-TB cultures as treatment duration increases, which allows identification of the evolution of ADR as drug concentrations fluctuate, as would occur in patients (18, 20). Application of RNA-sequencing (RNA-Seq) to these HFS-TB contents, in tandem with WGS, could allow us to “view” the “antibiotic resistance arrow of time” during the ADR process (21–23). Therefore, the present study was designed to identify the linezolid exposure target for sterilizing efficacy and suppression of ADR with no toxicity, as well as to identify ADR-related mutations based on WGS and to use transcriptomics to understand how the linezolid ADR arises.

## RESULTS

The linezolid MIC was 1 mg/liter, based on the 3 methods, on two different occasions each. The linezolid MIC in the presence of verapamil was 0.5 mg/liter. The MIC was 0.25 mg/liter with either thioridazine or reserpine, consistent with inhibition of constitutively expressed efflux pumps.

**FIG 1:**
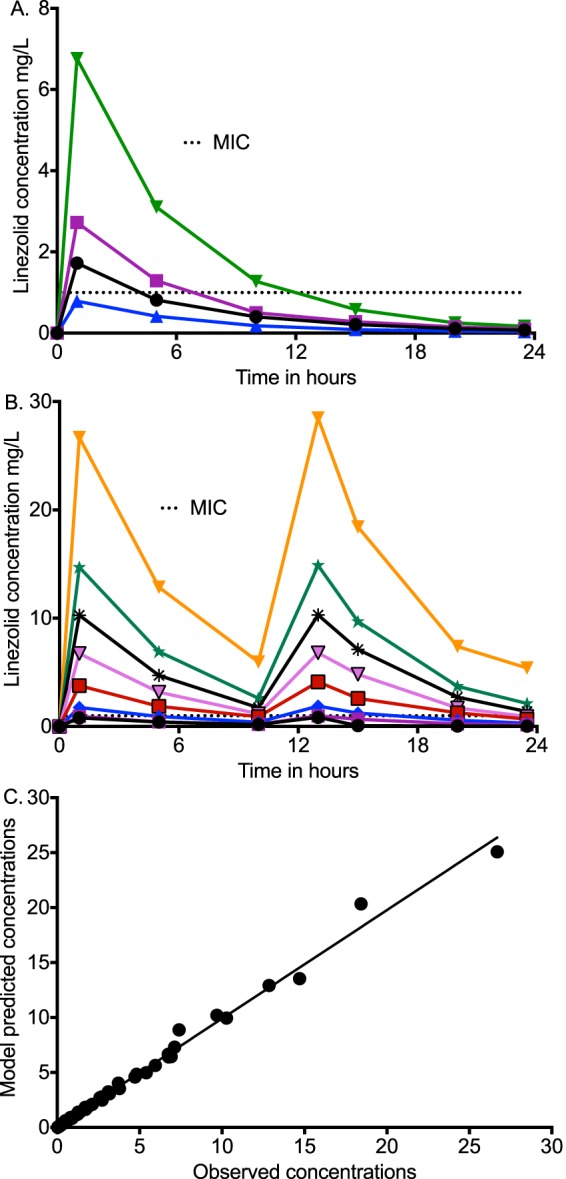
Pharmacokinetics of linezolid in the hollow fiber system tuberculosis model. (A) Concentration-time profile of linezolid achieved in regimens administered with a once-daily schedule, relative to MIC. (B) Concentration-time profile of linezolid versus MIC in the HFS-TB in which drug was administered every 12 h. (C) Observed versus one-compartment model predicted linezolid concentrations had a slope close to 0.99 ± 0.01 and thus close to unity (*r*^2^ > 0.99), which means no bias. Dotted lines represent the linezolid MIC for the strain used in the HFS-TB experiments.

The linezolid concentration-time profiles observed in the HFS-TB are shown in Fig. 1A and B. Pharmacokinetic modeling identified an elimination rate constant of 0.17 ± 0.01/hour and a half-life of 4.09 ± 0.32 h. The measured drug concentrations (observed) versus model prediction for the one-compartment model are shown in Fig. 1C.

**FIG 2:**
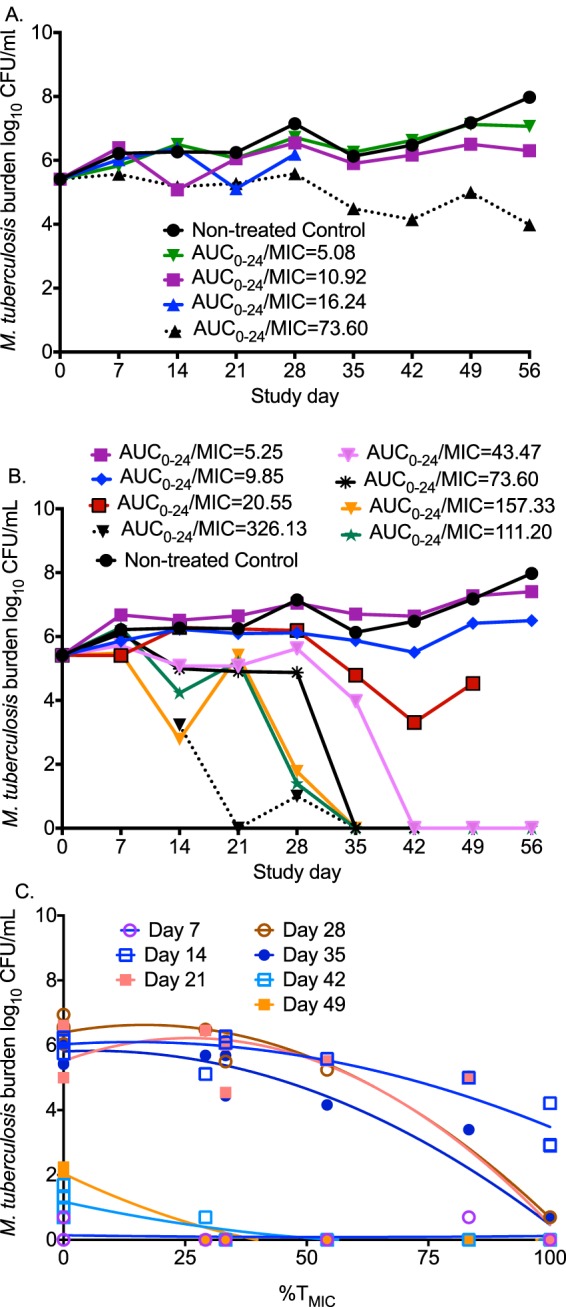
Linezolid kill curves for sterilizing activity and acquired drug resistance. (A) Time-kill curves for the once-daily dosing schedule show that an AUC_0–24_/MIC ratio of ≥73.6 killed to levels below day 0 bacterial burden (stasis line). (B) Time-kill curves with the twice-daily dosing schedule demonstrate bacterial burden below limits of detection in hollow fiber cartridges at high AUC_0–24_/MIC ratios. (C) The relationship between exposure and drug-resistant subpopulation is a system of changing U-shaped curves. The best fit for this quadratic function was with the percentage of time that the concentration persists above the MIC (%*T*_MIC_). A %*T*_MIC_ of 100 suppressed the linezolid-resistant subpopulation.

The bacterial burden at start of therapy was 5.41 log_10_ CFU/ml. The total M. tuberculosis bacterial burden in the nontreated HFS-TB grew at a rate of 0.03 ± 0.01 log_10_ CFU/ml/day over 56 days, confirming the semidormant state (21). The time-kill curves for each dosing schedule are shown in Fig. 2A and B. The figures show that over a period of more than a month, and starting around day 35 through day 56, progressively more regimens completely sterilized the systems. The time to negative culture based on linear regression (i.e., *x* intercept) for a treatment with a ratio of area under the concentration-time curve from 0 to 24 h (AUC_0–24_) to MIC of 73.6 was 68 days, that for the AUC_0–24_/MIC ratio of 111.2 was 46 days, and that for the next highest AUC/MIC ratio of 157.3 was 45 days.

Akaike information criterion (AIC) scores for the relationship between drug exposure and total microbial burden are shown in Table 1. The table shows that prior to replacement of most of the bacterial burden by the linezolid-resistant subpopulation in some HFS-TB experiments, AUC_0–24_/MIC ratio was the index linked to microbial kill. However, on day 21 the lowest AIC score was with the percentage of time that the concentration persisted above the MIC (%*T*_MIC_), which coincided with the highest increase in a transient linezolid-resistant subpopulation. The relationship between total M. tuberculosis burden and AUC_0–24_/MIC ratio on day 28 was effect (log_10_ CFU/ml) = (6.52 – 5.62) × AUC/MIC^3.41^/(AUC/MIC^3.41^ + 79.46^3.41^), *r*^2^ = 0.95, which calculates to an exposure associated with 80% of maximal kill (EC_80_) AUC_0–24_/MIC ratio of 119 and an upper 95% confidence interval bound of 152. The exposure associated with bacteriostasis (i.e., just holding the bacterial burden constant) was an AUC_0–24_/MIC ratio of 16.24. The exposure associated with 1.0 log_10_ kill was an AUC_0–24_/MIC ratio of 73.60.

**TABLE 1:** Akaike information criterion scores for microbial kill and acquired drug resistance^*c*^

Day	Microbial kill			ADR		
	AUC/MIC	*C*_max_/MIC	%*T*_MIC_	AUC/MIC	*C*_max_/MIC	%*T*_MIC_
7	**−13**	−13	NC^*a*^	**−19**	**−**19	−18
14	**−3**	−3	27	**−11**	−6	−4
21	4	4	**−4**	**11**	19	16
28	**−1**	−3	11	**−1**	11	7
35	**−1**	20	12	−2	8	**0.1**
42	17	23	**13**	NC^*b*^	NC^*b*^	NC^*b*^
49	23	29	**8**	−5	−6	**−14**
56	22	28	**2**	10	9	**−19**

a NC, nonconvergence.

b Drug-resistant subpopulation was below limits of detection on day 42.

c Boldface values indicate the lowest Akaike information criteria score, and therefore PK/PD index linked to microbial effect of either microbial kill or acquired drug resistance (ADR).

The AIC scores for the relationship between the linezolid-resistant subpopulation and exposure are also shown in Table 1. In the beginning, the lowest AIC scores were for AUC_0–24_/MIC ratio, but this switched to %*T*_MIC_ at later time points. The upright as well as inverted curves for the linezolid-resistant subpopulations for %*T*_MIC_ are shown in Fig. 2C, consistent with a series of U-shaped curves that change with time, described previously (20).

**FIG 3:**
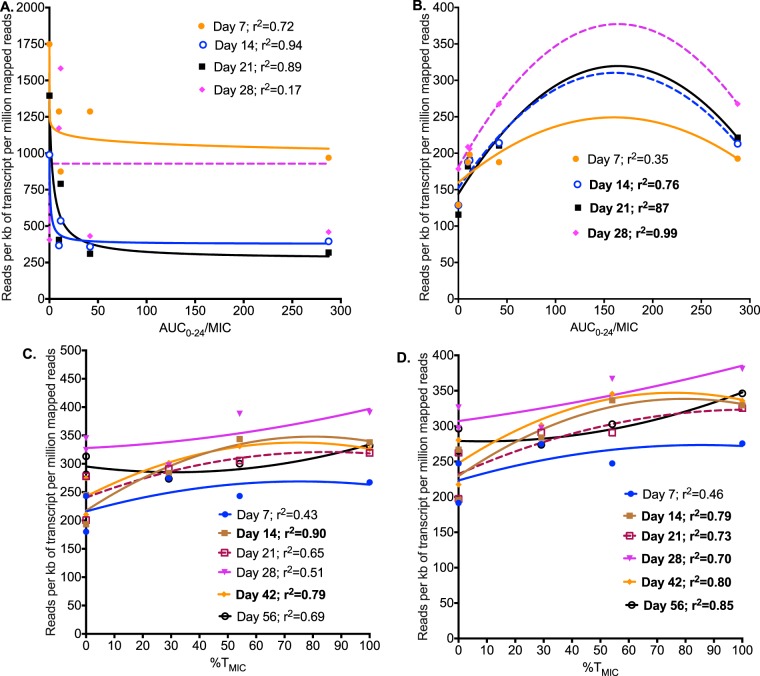
Linezolid resistance and Mycobacterium tuberculosis transcriptome. (A) The relationship between linezolid AUC_0–24_/MIC ratio and sequencing reads in protein synthesis pathway best fitted an inhibitory sigmoid *E*_max_ model, consistent with linezolid's mechanism of action. (B) The relationship between expression of all M. tuberculosis efflux pump genes and linezolid exposure best fitted the same quadratic function as that for exposure versus size of linezolid-resistant subpopulation. (C) One of three sets of specific efflux pump genes that were highly correlated with the size of the drug-resistant subpopulation was the daunorubicin-DIM transport membrane protein ABC transporter (*drrA*, *drrB*, and *drrC*). The relationship between RPKMs for these genes and linezolid exposure, as %*T*_MIC_, based on the quadratic function, was virtually the same as that for the linezolid-resistant subpopulation and exposure, especially on day 14 (shown in bold). (D) *mmpL9* demonstrated a similar pattern as well as the same relationship to the linezolid-resistant subpopulation.

**FIG 4:**
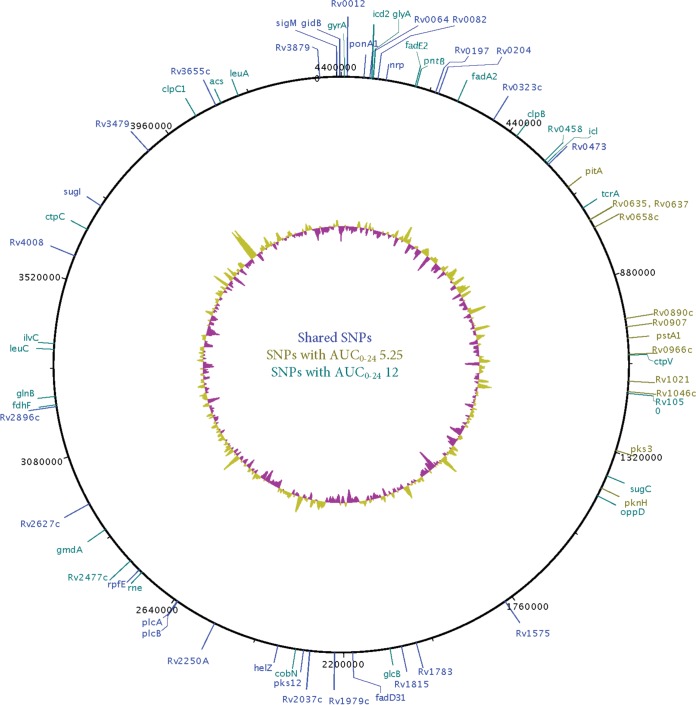
Mutation profile of linezolid-resistant M. tuberculosis. We show mutations present in nine linezolid-resistant M. tuberculosis isogenic strains compared to the wild type. A number of mutations, compared to wild type, occurred in genes encoding efflux pump/transporters (Rv0545c, Rv0930, Rv2477, and Rv3331), as well as in the *luxR* family transcriptional regulator Rv0890c.

The coverage and PHRED scores (data not shown) demonstrated that our sequencing was of good quality. Table S1 in the supplemental material shows all of the 138 differentially expressed genes (DEGs) identified on linezolid exposures compared to nontreated controls. The highest number of DEGs mapped to the M. tuberculosis protein synthesis, followed by efflux pumps. The relationship between AUC_0–24_/MIC ratio and protein synthesis gene reads per kilobase of transcript per million mapped reads (RPKMs) was examined using the inhibitory sigmoid maximum effect (*E*_max_) (Fig. 3A). The curve fits were good, except on day 28, consistent with the known mechanism of effect of linezolid. Efflux pump gene RPKMs were modeled using the same quadratic function for the drug-resistant subpopulation, with results shown in Fig. 3B. Figure 3C and D show the same modeling for the three most prominently upregulated efflux pump genes, *drrA*, *drrB*, and *drrC*, which encode ABC efflux pumps to multiple drugs (daunorubicin, tetracyclines, streptomycin, ethambutol, and rifampin), and *mmpL9*, encoding a resistance-nodulation-cell division family transporter. For efflux pump genes, there is clearly a series of U-shaped curves as was encountered with the linezolid-resistant subpopulation. The WGS revealed nonsynonymous mutations shown in Fig. 4. A detailed list of single nucleotide variants (SNVs) is shown in Table S2 in the supplemental material. Notable mutations included several in efflux pump/transporter genes (Rv0545c, Rv0930, Rv2477, and Rv3331) and the *luxR* family transcriptional regulator (Rv0890c).

**FIG 5:**
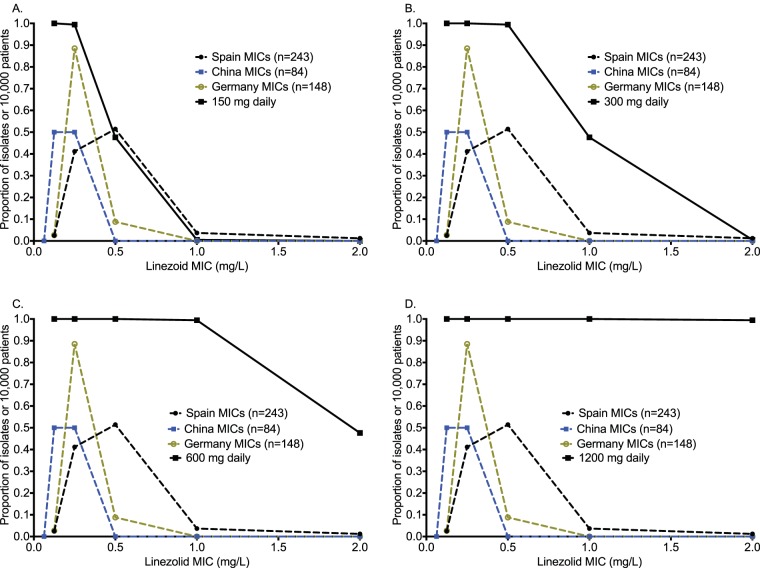
Target attainment probability of different linezolid doses in clinical trial simulations. (A) Target attainment probabilities of the linezolid 150-mg once-daily dose. (B) The target attainment probability of the 300-mg daily dose was >90% until an MIC of 1 mg/liter. (C) The dose of 600 mg per day performed well at all observed MICs, even with the MIC data set with the most leftward skew. (D) The 1,200-mg daily dose did not improve much on the 600-mg-per-day dose.

**FIG 6:**
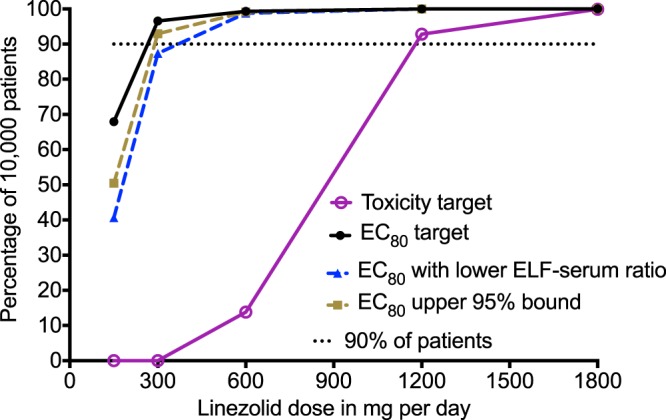
Cumulative fraction of response. The dose of 1,200 mg per day achieved the target AUC_0–24_ of 96 mg · h/liter associated with 50% mitochondrial inhibition in >90% of patients, consistent with the high adverse event rate, validating the approach. The dose of 600 mg a day achieved this AUC in <20% of patients. The dose of 300 mg a day achieved this AUC_0–24_ of 96 mg · h/liter in ∼0% of patients. In terms of efficacy, the dose of 300 mg a day achieved EC_80_ just shy of 90% of patients with the more stringent assumption on poorer ELF-to-serum penetration. The dose of 600 mg a day achieved the cumulative fraction of response that none of the higher doses improved much on, even with sensitivity testing such as lower ELF-to-serum ratio. Given that this is an AUC/MIC-linked effect, intermittent therapy (1,200 mg every other day) would also be as effective as a 600-mg/day dose.

Monte Carlo experiments in 10,000 adult tuberculosis patients faithfully recapitulated intended pharmacokinetic parameter estimates, as shown in Table 2. The AUC_0–24_ associated with administering the 600-mg dose in the simulations was 107.5 ± 30.16 mg · h/liter, which is similar to the value of 91.40 ± 39.3 mg · h/liter reported to the FDA for that dose (http://www.accessdata.fda.gov/drugsatfda_docs/label/2012/021130s028lbl.pdf). Thus, the exposures that we simulated mirror those achieved in the clinic. The target attainment probability for each of the doses for the EC_80_ target is shown in Fig. 5A to D. At the dose of 300 mg/day, the susceptibility breakpoint for linezolid against M. tuberculosis would be 1.0 mg/liter (i.e., the MIC at which <90% achieve target), while for 600 mg/day, it would be 2 mg/liter. The cumulative fraction of response (CFR) is shown in Fig. 6, which demonstrates that 300 mg/day would be optimal. Based on sensitivity analysis, whereby we either changed the assumptions of drug penetration into the lung or used the upper 95% confidence bounds of the EC_80_ AUC_0–24_/MIC ratio of 152 as target, 300 mg also did relatively well, but the 87.34% just missed the 90% target even when the lower epithelial lining fluid (ELF)-to-serum penetration ratio was assumed. The dose of 600 mg once a day consistently achieved a CFR of ≥99.3%. Finally, in Fig. 6, our simulations also addressed the likelihood of each of these doses achieving the threshold AUC_0–24_ value of 96 mg · h/liter associated with 50% inhibition of mitochondrial enzyme-related toxicity (9). The dose of 1,200 mg/day achieved the toxicity target in >90% of patients, and that of 600 mg/day achieved the target in just less than 20%, while that of 300 mg/day achieved this in ∼0% of patients.

**TABLE 2:** Pharmacokinetic parameter estimates as observed in patients and those from our simulations

Parameter	Value observed in:			
	435 patients		10,000-patient simulation	
	Mean	IIV^*a*^ (%)	Mean	Range
Clearance (liters/h)	6.0	46.6	5.8	2.03–18.2
Vol (liters)	54.53	25.9	54.4	42.1–69.9
Absorption constant (h^−1^)	0.58	40	0.59	0.1–9.7

a IIV, interindividual variability as percent coefficient of variation.

## DISCUSSION

The first important finding is that the AUC_0–24_/MIC ratio was the best driver for linezolid sterilizing effect efficacy. This has important practical implications for tuberculosis programs. It means that either once a day, every other day, or even twice a week will have the same efficacy as a twice-a-day therapy regimen, provided that the same cumulative dose is administered. Intermittent therapy is preferred and more practical for many programs, especially in resource-constrained settings. However, the final dosing schedule will depend on the drugs to be used in combination with linezolid, some of which may be optimized by intermittent dosing while others may not. As an example, drugs such as pyrazinamide and amikacin could be amenable to intermittent therapy (24, 25). Nevertheless, the utility of intermittent dosing and final dose will differ based on specific drugs used in the combination with linezolid.

Second, the standard 600-mg twice-per-day dose of linezolid is associated with a high rate of adverse events (3–5). We found that a dose of 600 mg/day was effective; indeed, even the dose of 300 mg/day (or 1,200 mg every 3 days) just missed the 90% proportion of patients that we use as a cutoff. Indeed, 300 mg/day would achieve the exposure associated with 1.0 log_10_ kill compared to baseline in >90% of patients. These results suggest that clinical trials should focus on comparing the efficacy of 300 mg/day (or 1,200 mg twice a week) with that of 600 mg/day (or 1,200 mg every alternate day) and not 600 mg twice a day. The proportion of patients achieving the linezolid concentration associated with toxicity just breached the 10% rate with 600 mg/day but was virtually zero at 300 mg/day. According to the work of Song et al., it is the trough (*C*_min_) linezolid concentration that is associated with toxicity; in that case, intermittent dosing schedules would be even more advantageous (26). However, the authors did not examine a potential role for AUC_0–24_ versus linezolid toxicity, which we did in our prior HFS-TB study that examined mitochondrial protein synthesis (9). On the other hand, linezolid AUC_0–24_ and *C*_min_ in patients could be colinear, so that the *C*_min_ could be a surrogate for AUC_0–24_.

Third, we caught acquired linezolid resistance in the act of occurring. There was an early but transient concentration-dependent increase in the linezolid-resistant subpopulation, best described by our quadratic model. There was a parallel early upregulation of efflux pump genes in the first 1 to 2 weeks, which demonstrated the same exposure-dependent system of U curves as in the linezolid-resistant subpopulation. With increased duration of therapy, efflux pump (27–32) gene mutations arose between months 1 and 2. This sequence of events is exactly what we have proposed in the “antibiotic resistance arrow of time model” and is consistent with findings in the first report of linezolid-resistant clinical isolates (1, 23, 33, 34). The *luxR* family transcriptional regulator (Rv0890c) mutation is also interesting: this gene is downregulated by tetracycline (http://tuberculosis.bu.edu/cgi-bin/GeneDetails.html?id=SRv0890c), as negative transcriptional regulators associated with antibiotic efflux would be. On the other hand, none of these linezolid-resistant isolates had mutations in the *rrl* or *rplC* genes, noted by others in the past. In the work of Richter et al. (1), alignment of all complete 23S rRNA gene and *rpL* gene sequences revealed no alterations between susceptible or linezolid-resistant strains but instead demonstrated a reduction in MIC with efflux pump inhibitors (1), consistent with our transcriptome and WGS findings.

Our study has some limitations. We used only a single M. tuberculosis strain in the HFS-TB experiments for identification of optimal exposures. Pharmacodynamic targets may change slightly when more bacterial strains are used in experiments. However, use of a single strain in this system has been highly predictive of optimal exposures in patients in the past (17). In addition, we incorporated the MIC distribution of many clinical strains in our Monte Carlo simulations, thereby taking into account the distribution of sensitivities of multiple strains.

In summary, we found that an intermittent dosing regimen for linezolid would be as effective as a daily dosing regimen. A dose of 300 mg/day or 1,200 mg twice a week should be examined by clinicians and compared to 600 mg/day or 1,200 mg every other day. These lower doses will likely reduce toxicity while continuing to optimize the sterilizing effect.

## MATERIALS AND METHODS

### Bacteria, materials, and reagents.

M. tuberculosis H37Rv was used in all the experiments, and cultures for sterilizing activity experiments were propagated as described previously (20). Linezolid was purchased from the Baylor University Medical Center pharmacy; verapamil, reserpine, and thioridazine were purchased from Sigma-Aldrich (St. Louis, MO), and hollow fiber cartridges were purchased from FiberCell (USA).

### Establishment of MIC.

The linezolid MIC was identified using the Etest and broth macrodilution methods. The MIC was also identified using the mycobacterial growth indicator tube (MGIT) assay (Becton Dickinson, Franklin Lakes, NJ) for linezolid alone, as well as in the presence of three different efflux pump blockers (verapamil at 40 mg/liter, reserpine at 10 mg/liter, or thioridazine at 1 mg/liter). These concentrations of the efflux pump blockers have been shown to have no effect on M. tuberculosis growth in the past (35).

### Linezolid hollow fiber dose-response and dose fractionation study.

The HFS-TB model for sterilizing effect has been described in detail previously (7, 20). Compared to serum, the linezolid 0- to 24-h area under the concentration-time curve (AUC_0–24_) values in epithelial lining fluid (ELF) were 3.3-fold higher in one study, while the average pairwise concentration comparisons were 8.4- ± 11.7-fold higher in another study; half-lives were similar in ELF and serum (13, 15). Fifteen HFS-TB units were inoculated with semidormant M. tuberculosis culture at pH 5.8. We mimicked the half-life of 4 h in adult patients (36) and AUC_0–24_ penetration of 3.3-fold in ELF. The HFS-TB units were treated with linezolid to achieve free drug AUC_0–24_s of 5, 10, 20, 40, and 80, administered on either a once- or a twice-daily schedule, and AUC_0–24_ exposures of 111, 157, and 326 were administered on a twice-daily schedule. We sampled the central compartments of each HFS-TB at 0, 1, 5, 10, 15, 20, and 23.5 h after the first dose to confirm that the intended concentration-time profiles were achieved, and we measured concentrations using assays described previously (9, 10). The peripheral compartment of each HFS-TB was sampled on days 0, 3, and 7 and then every 7 days for 2 months. A portion of the sample was immediately added to tubes prepared for RNA protection and extraction. Next, a portion of the samples was washed twice with normal saline to remove any drug carryover as described previously and then serially diluted (7, 37). Cultures were inoculated on Middlebrook 7H9 agar supplemented with 10% oleic acid-dextrose-catalase (OADC) at 37°C under 5% CO_2_, and colonies were counted after 21 days of incubation. To determine the linezolid-resistant subpopulation sizes, agar was supplemented with 3 times the linezolid MIC and incubated for up to 6 weeks before colonies were counted. Some of the linezolid-resistant isolates were picked, regrown to a large biomass, and then processed for WGS.

### WGS of linezolid-resistant isolates.

DNA was extracted from nine M. tuberculosis isolates resistant to 3 times the MIC (38). Sequencing libraries were prepared using the Kapa Biosystems Hyper kit (KK8504). After size selection, adapter-ligated genomic libraries were amplified using 4 PCR cycles and cleaned using XP beads. A 9 pM concentration of each library was used for sequencing on a HiSeq 2500 PE100 (paired-end 100-bp) lane. The sequencing reads were sorted based on attached barcodes using SAMtools (http://samtools.sourceforge.net/), processed to remove adapter artifacts, and deconvoluted into their constituent isolates. Reads with no identifiable barcode or with a barcode containing one or more ambiguous base calls were excluded. CLC Genomics workbench (v9.5.2) was used to determine the read quality, nucleotide content, and sequence redundancy. Finally, reads were aligned to the reference M. tuberculosis genome (NC_000962) and single nucleotide variants (SNVs) were made compared to the wild type.

### RNA extraction and sequencing of M. tuberculosis from hollow fiber samples.

RNA extraction from each of the HFS-TB samples and sequencing were performed using steps that we have described previously (21). Quality control of the reads and alignment to the M. tuberculosis reference genome (NC_000962) were performed using the CLC Genomics workbench (v9.5.2). The expression of various genes was calculated in terms of reads per kilobase of transcript per million mapped reads (RPKM). RPKM values were normalized using the quantile method, and multigroup comparison was performed.

### Bioinformatic analyses.

Differentially expressed genes (DEGs) were defined based on comparison of expression in the linezolid-treated systems with that in nontreated controls with ≥1.5-fold expression change and a Bonferroni posttest correction-adjusted *P* of <0.05. We used the KEGG pathway search and mapping tool (http://www.genome.jp/kegg/tool/map_pathway2.html) to map the DEGs to M. tuberculosis pathways. In the next step, we generated gene modules for the pathways identified, using all genes in the identified pathways. In addition, the relationship between drug exposure and RPKM values was examined using either the inhibitory sigmoid *E*_max_ model or our quadratic function for resistance modeling (14, 20).

### Pharmacokinetic-pharmacodynamic modeling.

The measured linezolid concentrations were modeled using the ADAPT 5 program (Biomedical Simulations Resource, University of Southern California), using steps described previously (7, 39). We used the inhibitory sigmoid *E*_max_ model to determine the relationship between exposure and total M. tuberculosis CFU per milliliter. In order to select for the pharmacokinetics/pharmacodynamics index linked to microbial kill, total bacterial burden versus exposure (either free drug AUC_0–24_/MIC ratio, percentage of time that concentration persisted above MIC [%*T*_MIC_], or peak concentration to MIC [*C*_max_/MIC] ratio or trough/MIC ratio) was examined for best fit to the inhibitory sigmoid *E*_max_ model, and the best index was chosen using Akaike information criterion (AIC) scores. We used the chosen model to calculate the exposure associated with 80% of maximal kill (EC_80_). For ADR, we utilized the quadratic function of Gumbo et al. (45) for the relationship between linezolid exposure and the size of the drug-resistant subpopulation; the best pharmacokinetics/pharmacodynamics index was also chosen using AIC scores.

### Dose selection for optimal effect and minimal toxicity.

In order to identify the minimal dose for linezolid sterilizing effect best able to achieve or exceed the EC_80_ in >90% of patients, we performed Monte Carlo simulations of 10,000 adult tuberculosis patients. The population pharmacokinetic parameter estimates input into subroutine PRIOR were those identified by Abe et al. (40) in 435 patients, shown in Table 2 (40). The linezolid ELF-to-serum AUC_0–24_ penetration ratio of 3.29 was also taken into account, as was the 30% protein binding (15). The target attainment probability, which is how well a dose of 150 mg, 300 mg, 600 mg, or 1,200 mg would achieve the EC_80_ in the lung of tuberculosis patients at each MIC, was then calculated. We utilized three different MIC distributions from Spain, China, and Germany (466 clinical isolates) (41–43). The cumulative fraction of response (CFR) for each dose was then summated over the MIC distribution. We performed two sensitivity analyses. The first test was to identify the CFR if the EC_80_ was at the upper 95% confidence interval value. The second tested the assumption that linezolid did not penetrate with an ELF-to-serum ratio of 3.29 but instead with a value of 1.52 as identified by Boselli et al. (15, 44).

We have identified the threshold AUC_0–24_ value of 96 mg · h/liter as being associated with 50% inhibition of mitochondrial enzyme-related toxicity (9). For each dose studied, we calculated the proportion of patients who would achieve or exceed the serum AUC_0–24_ of 96 mg · h/liter. We set a 20% threshold as an unacceptably high proportion of patients achieving concentrations associated with toxicity. For external validation, we determined the proportion that would achieve this with the standard dose of 1,200 mg a day, in order to relate to what is observed in the clinic.

## Supplementary Material

Supplemental material
