# Supplementary material for: Linezolid Dose That Maximizes Sterilizing Effect While Minimizing Toxicity and Resistance Emergence for Tuberculosis
Source: Antimicrob Agents Chemother. 2017 Jul 25;61(8):e00751-17. doi: 10.1128/AAC.00751-17 (PMC5527615; doi:10.1128/AAC.00751-17)
Supplement: Supplemental material [file supp_61_8_e00751-17__index.html]

Supplemental material 

# Linezolid Dose That Maximizes Sterilizing Effect While Minimizing Toxicity and Resistance Emergence for Tuberculosis

## Supplemental material

- Supplemental file 1 -

  Supplemental Table S1

  XLSX, 69K
- Supplemental file 2 -

  Supplemental Table S2

  XLSX, 12K
